# Arrested crossover precursor structures form stable homologous bonds in a *Tetrahymena* meiotic mutant

**DOI:** 10.1371/journal.pone.0263691

**Published:** 2022-02-16

**Authors:** Miao Tian, Kazufumi Mochizuki, Josef Loidl

**Affiliations:** 1 Department of Chromosome Biology, Max Perutz Labs, University of Vienna, Vienna, Austria; 2 IGH (Institute of Human Genetics), Montpellier, France; Tulane University Health Sciences Center, UNITED STATES

## Abstract

Meiotic DNA double-strand breaks produce reciprocally exchanged DNA strands, which mature into chiasmata that hold homologous chromosomes together as bivalents. These bivalents are subsequently separated in the first meiotic division. In a mutant lacking the newly identified *Tetrahymena* gene *APRO1* (Anaphase promoting 1), meiosis is arrested by the end of prophase. Mature chiasmata are not formed but bivalents are connected via a molecular precursor structure. In-depth analysis of this arrested intermediate structure may help to elucidate the noncanonical molecular recombination pathway in *Tetrahymena*.

## Introduction

Meiotic crossovers form the basis of genetic recombination, which contributes to diversity in sexual progeny through generating novel combinations of traits. These can become fixed in species via natural selection. Crossovers are initiated by programmed DNA double-strand breaks (DSBs; see [1]). The 5´ strands of DNA ends flanking DSBs become resected and then 3´ single-stranded DNA tracts invade double-stranded DNA molecules. Stand invasion enables the search for complementary base sequences, i.e. homologous chromosome regions. At these sites, the invading strand can initiate reciprocal recombination by crosswise ligation with the corresponding strand from the homolog. Remaining single-stranded gaps at recombination sites are filled by DNA repair synthesis, resulting in a microscopically visible chiasma. Chiasmata connect pairs of homologous chromosomes (then called a bivalent) until the first meiotic division, when the diploid somatic chromosome complement is reduced to a single gametic chromosome set.

**Fig 1:**
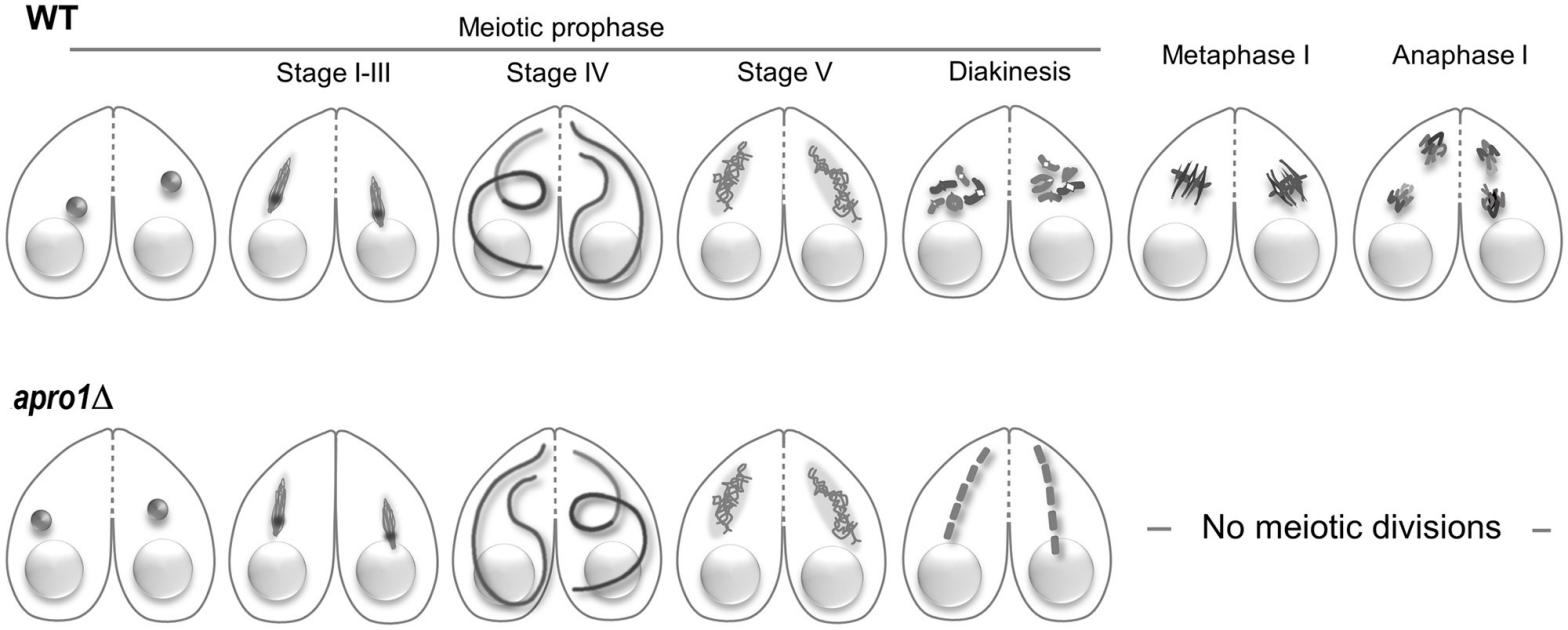
Schematic representation of meiosis in the wild type and *apro1*Δ mutant. Wild-type meiosis is characterized by transient elongation of the germline nucleus during prophase. At metaphase I, condensed bivalents assemble at the nuclear equator. In the mutant, early prophase is normal, but by the end of prophase, condensed bivalents are aligned in tandem along the elongated nucleus; meiotic divisions do not take place.

In the ciliate *Tetrahymena thermophila*, DSBs not only initiate crossing over but also trigger elongation of the meiotic nucleus to about twice the length of the cell (Fig 1). The tight parallel arrangement of chromosome arms within the tubular nucleus promotes prealignment of the homologs (see [2]). While in most eukaryotes, DSBs are processed along at least two pathways leading to interfering (mutually suppressing) or noninterfering crossovers (along with noncrossover outcomes), *Tetrahymena* seems to use a single merged pathway to generate crossovers (see [3]).

Here we report a gene, *APRO1* (Anaphase promoting 1), in whose absence meiosis is arrested by the end of prophase, with incomplete crossovers and bivalents adopting an unusual tandem arrangement. The accumulation of crossover precursors in the deletion mutant (*apro1*Δ) offers the possibility to analyze the molecular nature of an intermediate stage in the *Tetrahymena* crossover pathway.

## Methods

We used a null mutant of *APRO1* (TTHERM_00112830, http://www.ciliate.org/ [4], which was previously produced by the deletion of a 707-bp sequence including most of the open reading frame [5]. To generate an *apro1*Δ *spo11Δ*D double mutant, a plasmid carrying a ~750 bp sequence flanking the *APRO1* open reading frame and a selectable *CHX* (cycloheximide resistance) marker was ligated by Gibson assembly, using primers #1 to #4 (S1A Fig). The resulting knockout plasmid was linearized and then introduced into *spo11*Δ cells by biolistic transformation [6]. (For the generation of *spo11*Δ cells see [7]). Gene knockout was confirmed by qPCR, and loss of gene expression by reverse transcription PCR using primers #5 and #6 (S1 Fig).

Cells were grown under standard conditions, and starved cells of complementary mating types were mixed to induce conjugation (= cell mating) and meiosis [8]. Different fixation and staining protocols were applied: To follow changes in nuclear shape, conjugating cells were fixed in formaldehyde at various time points and stained with DAPI [9]. Optical slices of cells were photographed and images were deconvolved and 2D-projected, as described in [7]. To detect Apro1, strains expressing C-terminally EGFP-tagged Apro1 [10] were used. Dmc1 immunostaining of high-detergent-fixed cells was done according to established protocols [11]. For the analysis of bivalents, cells were fixed in Schaudinn´s fixative, spread on slides and stained with Giemsa [12].

To detect of recombination-related DNA synthesis, conjugating cells were fed with the thymidine analog bromodeoxyuridine (BrdU) at 2 h or 3 h after meiosis induction and then harvested at 4 h 15 min after meiosis induction. BrdU incorporation was detected in fixed chromosomes using a Rat anti-BrdU antibody (for details see [13]).

Artificial DNA damage was induced by exposure of conjugating cells to short-wave ultraviolet (UV) radiation (240 nm, 20 Joule/m^2^) 2 h after meiosis induction [14].

For pulsed-field electrophoresis (PFGE), genomic DNA was embedded in low melting point agarose plugs. DNA was separated by running in 1% agarose in 0.5× TBE buffer at 6 V/cm and 14°C for 14 h with 60-sec pulses, 10 h with 90-sec pulses and 1 h with 120-sec pulses. DSB-dependent fragments were detected by Southern hybridization with a probe specific to germline chromosomes. For details of the method see [15].

## Results

*APRO1* had been knocked out because its expression in early conjugating (= meiotic) cells suggested a function in meiosis or the post-meiotic stages of sexual reproduction [5]. (For the expression profile of TTHERM_00112830 (*APRO1*) see http://tfgd.ihb.ac.cn/ [16]. It was found that the mutant did not produce meiotic progeny. While the previously coined name of the gene referring to a function in anaphase promotion [2] does not well apply to the actual mutant phenotype as shown below, it is retained to avoid confusion.

**Fig 2:**
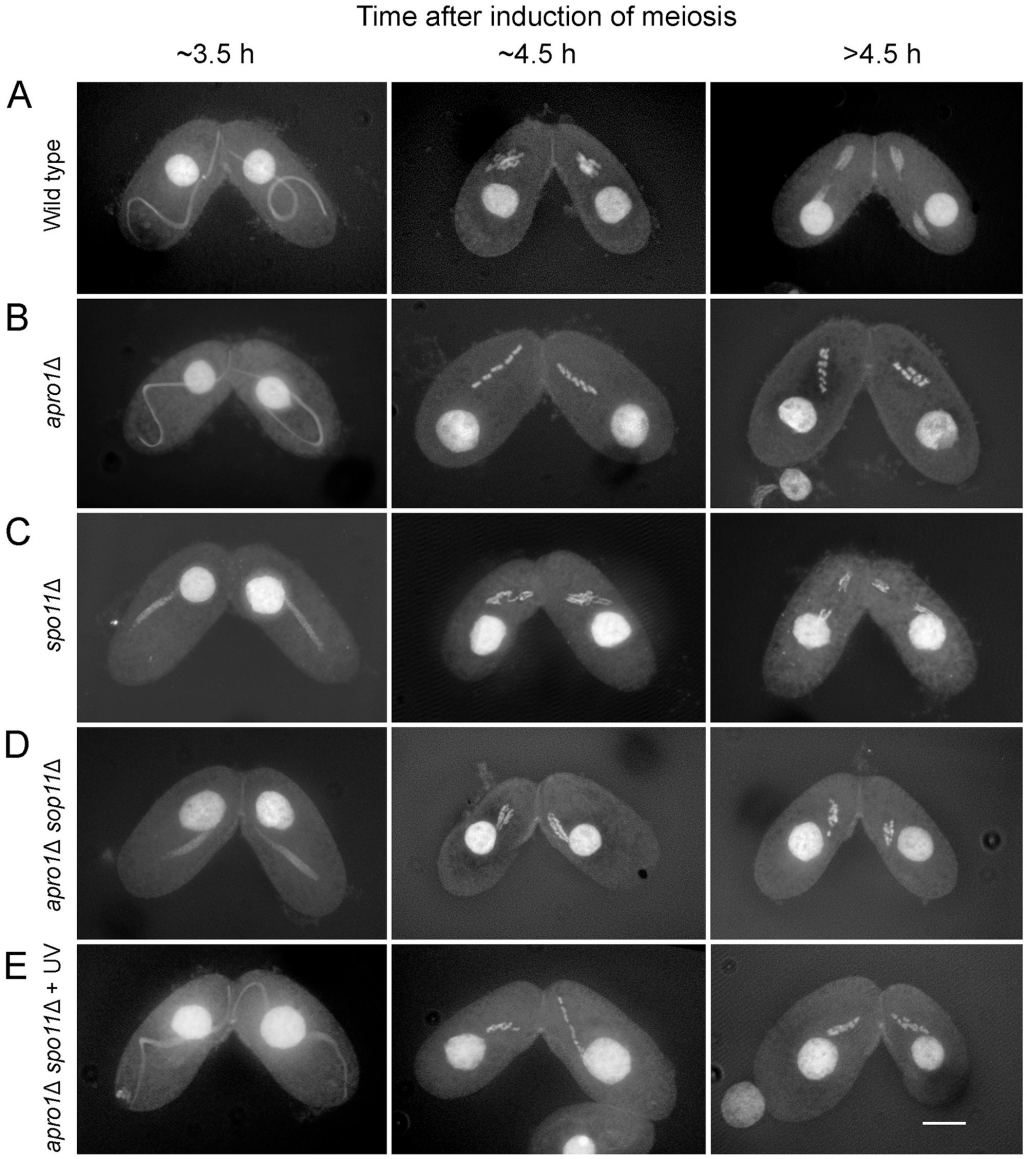
Meiosis in the wild type and in mutants. A. Pairs of mating wild-type cells undergoing synchronous meioses with transient elongation of the meiotic germline nucleus (~3.5 h after induction of meiosis) followed by the arrangement of bivalents in a metaphase plate (~4.5 h) and the first meiotic division. B. The *apro1*Δ mutant shows a characteristic tandem arrangement of five condensed bivalents and does not enter anaphase I. C. No nuclear elongation occurs in the *spo11*Δ mutant due to the absence of DSBs. D. Neither nuclear elongation nor tandem arrangement of chromosomes occurs in the *apro1*Δ *spo11*Δ double mutant. E. If nuclear elongation is restored by artificial DNA damage in the *apro1*Δ *spo11*Δ double mutant, the tandem arrangement of univalents is also restored. Bar: 10 μm.

**Fig 3:**
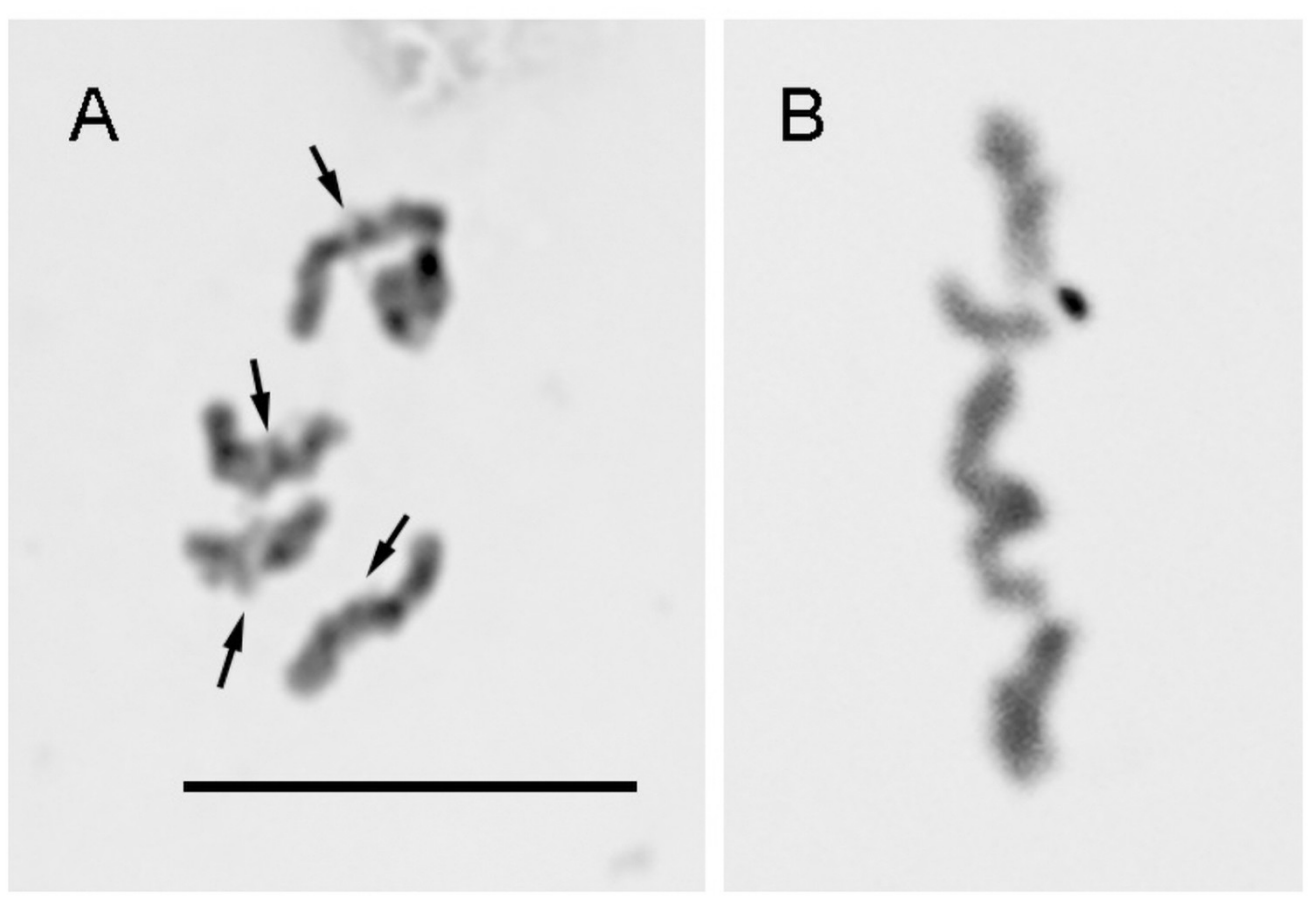
Giemsa-stained bivalents. A. In the wild type, kinetochores (arrows) are often seen to project from chromosomes, indicating pulling by spindle microtubuli. B. This was never observed in the mutant when chromosomes were similarly compacted. Bivalents are not strictly arranged in tandem due to the breaking of the nuclear membrane caused by this method. Bar: 10 μm.

Here, we studied the mutant in more detail and found that the *apro1*Δ mutant showed abnormal meiotic behavior: Whereas early prophase progresses normally, at the end of prophase, condensed bivalents were aligned in tandem and meiotic divisions did not take place (Figs 1 and 2). These bivalents do not show projecting kinetochores, which in the wild type indicate the attachment of microtubules of the intranuclear division spindle (Fig 3). To see whether tandem chromosome arrangement was a consequence of pair formation, we produced a double mutant of *apro1* with *spo11*. (The *spo11*Δ mutant fails to form homologous pairs and does not undergo nuclear elongation due to the absence of DSBs—[7]). The tandem arrangement of bivalents was not seen in the *apro1*Δ *spo11*Δ double mutant. However, when nuclear elongation was restored in the double mutant by UV-induced DNA damage [14], the tandem arrangement (of univalents) was partially restored (Fig 2). This result suggests that the tandem arrangement occurs independently of homologous pair formation. It is rather a consequence of the spatial restriction within the elongated nucleus caused by a loss of coordination between the processes of chromosome compaction and exit from nuclear elongation in the absence of Apro1.

**Fig 4:**
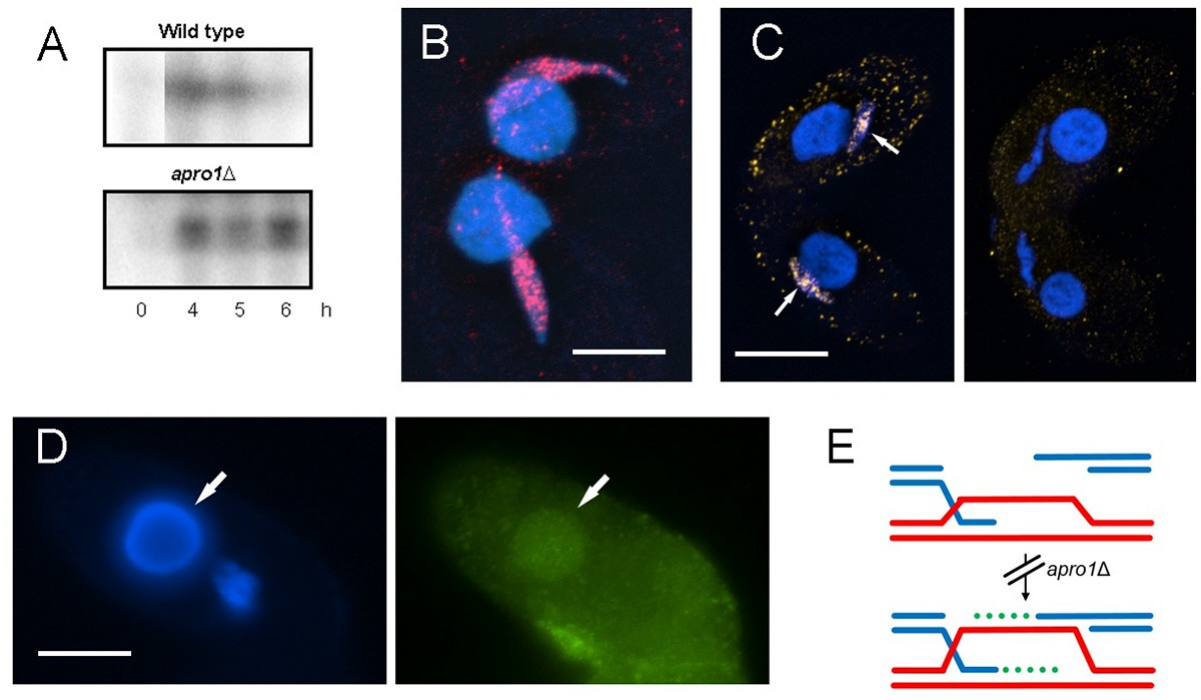
A. PFGE shows the transient appearance of a band representing DSB-dependent chromosome fragments in the wild type. In the *apro1*Δ mutant chromosome fragments persist. (Lanes from additional timepoints of the wild type were removed for better comparison.) B. Dmc1 (red) localizes to chromatin in the *apro1*Δ mutant. C. BrdU (yellow) is incorporated during repair-related DNA synthesis in the wild type (left, arrows), but is not visibly incorporated in the mutant (right). D. EGFP-tagged Apro1 (green) localizes to the somatic nucleus (arrow). Bars: 10 μm. E. Model showing a hypothetical arrested recombination intermediate in the *apro1*Δ mutant. In the wild type, invasion of a homologous DNA tract by two ends of a broken DNA is followed by gap-filling repair synthesis (green dots). In *apro1*Δ, a single DNA end invading a homologous region is not elongated by newly synthesized DNA, but the limited heteroduplex is sufficient to hold homologs together. The other end does not invade, explaining the persistent DNA fragments seen in PFGE.

To study the progress of DSB formation and repair in the *apro1*Δ mutant, PFGE was used to monitor the presence of DSB-dependent chromosome fragments. While in the wild type these fragments appear transiently during meiotic prophase, their persistence in the mutant suggests that DSBs are not or incompletely repaired (Fig 4A). To test whether single-strand resection occurs at DSB sites, we looked for the presence of Dmc1 in meiotic nuclei because Dmc1 associates preferentially with the single-stranded DNA ends flanking DSBs [17]. We immunostained cells that had been fixed in the presence of a high concentration of detergent–a condition, which removes free protein and preserves only chromatin-bound Dmc1 [11]. Strong Dmc1 foci were found, suggesting that normal strand resection occurs at DSBs (Fig 4B). Next, we aimed to determine whether the subsequent steps in meiotic DSB processing, namely homologous strand invasion and gap-filling repair synthesis, also occur. For this, BrdU was added to meiotic cells prior to fixation, and its incorporation was monitored by immunostaining the cells after fixation. Surprisingly, and in contrast to the wild type, BrdU was not detected in 200 nuclei analyzed at the appropriate stage (Fig 4C), suggesting that repair synthesis is either absent or reduced to below the level of detection. This observation was unexpected because it means that an intermediate recombination structure strong enough to hold homologs together exists in the absence of mature crossovers and chiasmata.

The phenotype of the *apro1*Δ mutant, including the linear arrangement of bivalents and the reduction or complete absence of repair DNA synthesis, closely resembles the phenotype caused by a mutation in the transcription factor *E2FL1* [18]. Moreover, Apro1 protein localizes exclusively to the somatic but not to the germline nucleus of meiotic cells (Fig 4D), suggesting that it is also involved in gene regulation.

## Discussion

In *Tetrahymena* wild-type meiosis, condensed bivalents are formed by the time the meiotic nucleus has exited from the elongated state. However, in the *apro1*Δ mutant, condensed bivalents become visible when the nuclei are still elongated. The absence of projecting kinetochores in these bivalents (Fig 3) indicates that meiosis is arrested before the onset of the first division. Moreover, bivalents are arranged in tandem within the nucleus. This suggests that this arrest also involves the failure to restore the elongated state of the prophase nucleus to a more rounded shape that can accommodate metaphase I bivalents side-by-side (as seen in the wild type).

In addition to these cytological anomalies, the absence of Apro1 causes a defect in DSB processing. Reduced or absent DNA repair synthesis leads to the formation of an indeterminable number of connections between homologs that are not mature crossovers but are sufficient to stabilize bivalents. These recombination intermediates are presumed to consist of single end invasions that form heteroduplexes, which are not or only slightly extended by newly synthesized DNA. The second end would not invade the homolog and would be visible by PFGE as DNA fragment. Fig 4E shows a model of the arrested recombination intermediate.

Localization of Apro1 to the somatic nucleus in conjugating (= meiotic) cells suggests that it is a gene regulatory factor. In fact, the expression profile of *APRO1* is very similar to that of the transcription factor *E2FL1* (http://tfgd.ihb.ac.cn/ [19]), and its expression is upregulated by ~10-fold in the absence of E2fl (Wei Miao, pers. commun.). In addition, the molecular and cytological phenotypes of *apro1* and *e2fl1* deletion mutants are practically identical [20]. Thus, together with Dpl2 [18], E2fl and Apro1 may co-regulate gene expression during conjugation. It is possible that the apparently unrelated phenotypes of *apro1*Δ result from the combined misregulation of two or more genes: Failure to incorporate BrdU may be due to the incomplete processing of recombination intermediates, whereas the tandem arrangement of bivalents and failure to produce anaphase I tension may be caused by a failure to reorganize microtubules (from stretching the nucleus to forming the division spindle [21]). Therefore, the factors directly responsible for meiotic arrest with anomalous bivalents remain undefined.

The *apro1*Δ mutant provides a unique tool to observe a crossover intermediate stage. It is hoped that together with ongoing efforts toward mapping meiotic DSB hotspots in *Tetrahymena* it will allow us to elucidate the molecular nature of the elusive recombination intermediate of *Tetrahymena*´s unconventional crossover pathway.

## Supporting information

S1 Fig Generation and testing of apro1Δ spo11Δ double mutant cells. (PPTX)

S2 Fig Raw versions of gel blots shown in Fig 4A. (PPTX)
