# Supplementary material for: Arrested crossover precursor structures form stable homologous bonds in a Tetrahymena meiotic mutant
Source: PLoS One. 2022 Feb 16;17(2):e0263691. doi: 10.1371/journal.pone.0263691 (PMC8849441; doi:10.1371/journal.pone.0263691)
Supplement: S1 Fig — (PPTX) [file pone.0263691.s001.pptx]

## Slide 1
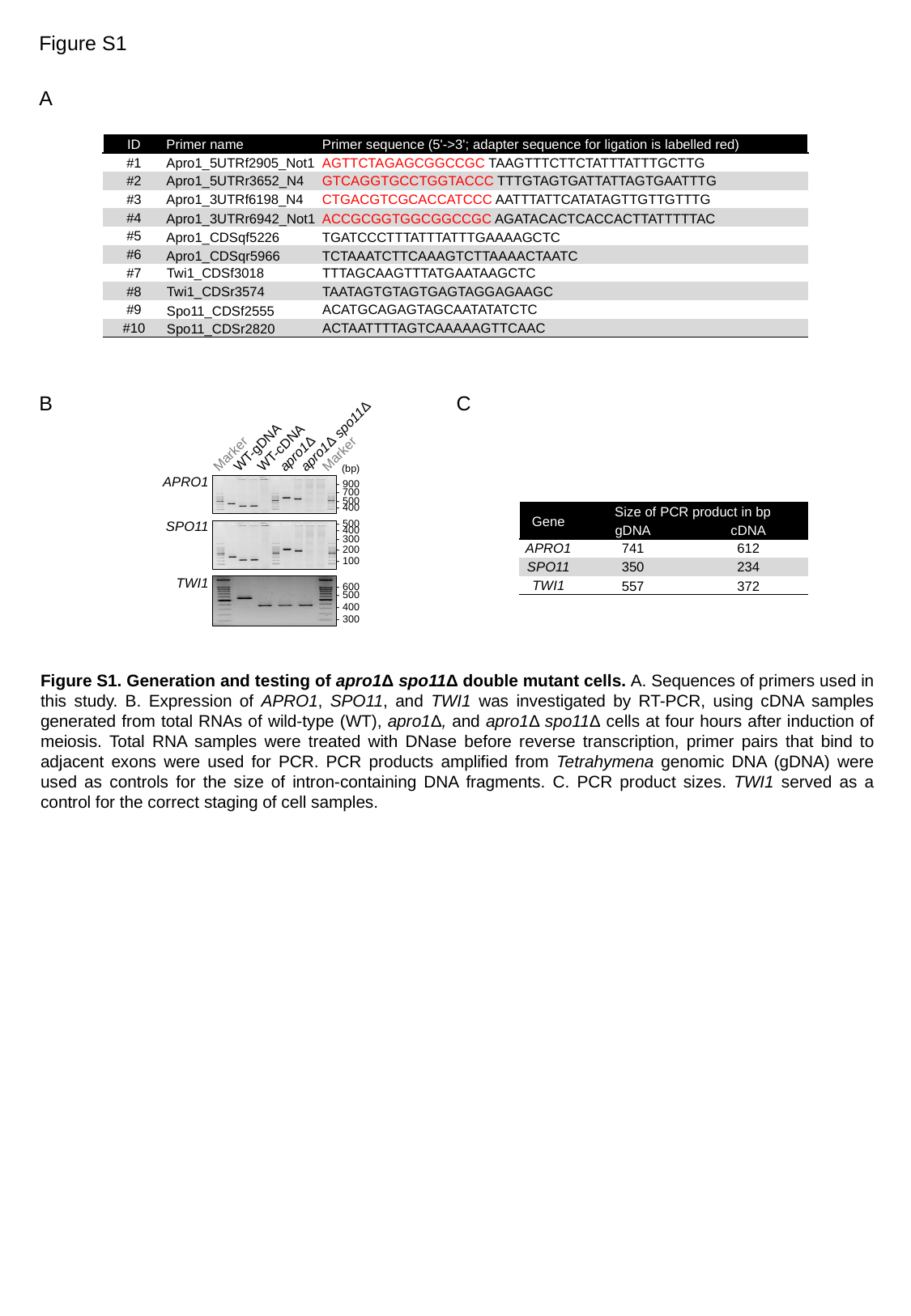

Figure S1
A
| ID | Primer name | Primer sequence (5'->3'; adapter sequence for ligation is labelled red) |
| --- | --- | --- |
| #1 | Apro1\_5UTRf2905\_Not1 | AGTTCTAGAGCGGCCGC TAAGTTTCTTCTATTTATTTGCTTG |
| #2 | Apro1\_5UTRr3652\_N4 | GTCAGGTGCCTGGTACCC TTTGTAGTGATTATTAGTGAATTTG |
| #3 | Apro1\_3UTRf6198\_N4 | CTGACGTCGCACCATCCC AATTTATTCATATAGTTGTTGTTTG |
| #4 | Apro1\_3UTRr6942\_Not1 | ACCGCGGTGGCGGCCGC AGATACACTCACCACTTATTTTTAC |
| #5 | Apro1\_CDSqf5226 | TGATCCCTTTATTTATTTGAAAAGCTC |
| #6 | Apro1\_CDSqr5966 | TCTAAATCTTCAAAGTCTTAAAACTAATC |
| #7 | Twi1\_CDSf3018 | TTTAGCAAGTTTATGAATAAGCTC |
| #8 | Twi1\_CDSr3574 | TAATAGTGTAGTGAGTAGGAGAAGC |
| #9 | Spo11\_CDSf2555 | ACATGCAGAGTAGCAATATATCTC |
| #10 | Spo11\_CDSr2820 | ACTAATTTTAGTCAAAAAGTTCAAC |
B
C
Marker
apro1Δ spo11Δ
Marker
WT-gDNA
WT-cDNA
apro1Δ
(bp)
APRO1
- 900
- 700
- 500
- 400
| Gene | Size of PCR product in bp | |
| --- | --- | --- |
| | gDNA | cDNA |
| APRO1 | 741 | 612 |
| SPO11 | 350 | 234 |
| TWI1 | 557 | 372 |
- 500
SPO11
- 400
- 300
- 200
- 100
TWI1
- 600
- 500
- 400
- 300
Figure S1. Generation and testing of apro1Δ spo11Δ double mutant cells. A. Sequences of primers used in this study. B. Expression of APRO1, SPO11, and TWI1 was investigated by RT-PCR, using cDNA samples generated from total RNAs of wild-type (WT), apro1Δ, and apro1Δ spo11Δ cells at four hours after induction of meiosis. Total RNA samples were treated with DNase before reverse transcription, primer pairs that bind to adjacent exons were used for PCR. PCR products amplified from Tetrahymena genomic DNA (gDNA) were used as controls for the size of intron-containing DNA fragments. C. PCR product sizes. TWI1 served as a control for the correct staging of cell samples.
