# Supplementary material for: Arrested crossover precursor structures form stable homologous bonds in a Tetrahymena meiotic mutant
Source: PLoS One. 2022 Feb 16;17(2):e0263691. doi: 10.1371/journal.pone.0263691 (PMC8849441; doi:10.1371/journal.pone.0263691)
Supplement: S2 Fig — (PPTX) [file pone.0263691.s002.pptx]

## Slide 1
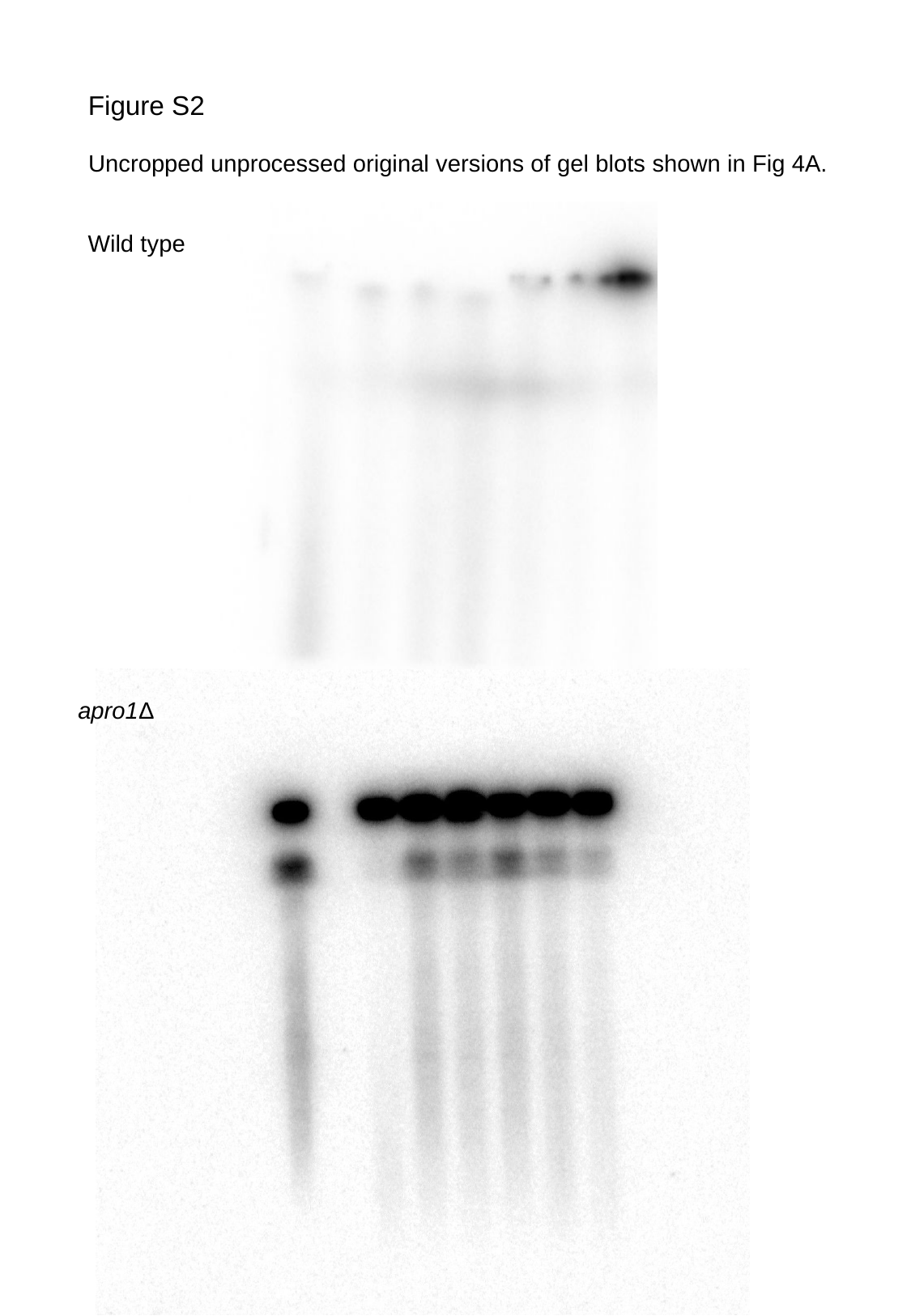

Figure S2
Uncropped unprocessed original versions of gel blots shown in Fig 4A.
Wild type
apro1Δ
